# *Cryptosporidium spp., Giardia duodenalis* and *Toxoplasma gondii* detection in fresh vegetables consumed in Marrakech, Morocco

**DOI:** 10.4314/ahs.v20i4.19

**Published:** 2020-12

**Authors:** Salma Berrouch, Sandie Escotte-Binet, Yassine Amraouza, Pierre Flori, Dominique Aubert, Isabelle Villena, Jamaleddine Hafid

**Affiliations:** 1 Laboratory of Food, Environment and Health, Faculty of Sciences and Techniques, Cadi Ayyad University, Marrakech, Morocco; 2 Laboratory of Parasitology-Mycology, ESCAPE EA 7510, SFR CAP SANTE, University of Reims Champagne-Ardenne, and CHU Reims, Hospital Maison Blanche, National Reference Centre of Toxoplasmosis, Reims, France; 3 Laboratory of Infectious Agents- Parasitology section- GIMAP, Faculty of Medicine, Saint-Etienne, France

**Keywords:** Fresh vegetables, protozoan parasites, Marrakech, qPCR

## Abstract

**Background:**

Protozoan parasites such as *Toxoplasma gondii, Giardia duodenalis*, and *Cryptosporidium spp.*, can be transmitted to humans via accidental consumption of contaminated water, fresh produce and foodstuffs. There is a lack of epidemiological data about these pathogens in Morocco. Hence the aim of this study, which is the determination of their prevalence in some leafy greens and root vegetables sold in Marrakech.

**Methods:**

A total of 132 vegetable samples including carrot, coriander, lettuce, parsley and radish were purchased monthly from three different markets in Marrakech from March 2017 to January 2018, pre-treated and subjected to microscopic and molecular analyses.

**Results:**

Of the 132 samples of vegetables analyzed by qPCR, the overall rate of protozoan was 21.21% (28/132); 22 samples were found to be contaminated with *T. gondii*, 6 with *G. duodenalis*, and none was positive for *C. parvum*/*hominis*. Whereas, modified Ziehl-Neelsen staining allowed the detection of *Cryptosporidium spp*. in 3% (4/132) of examined samples.

**Conclusion:**

This survey on the presence of protozoan parasites in fresh vegetables revealed that vegetables sold in Marrakech are contaminated by these protozoan parasites, as it showed that leafy green vegetables were more susceptible for parasitic contamination than root ones.

## Introduction

*Giardia duodenalis* and *Cryptosporidium spp.* are enteric water-borne parasites pathogenic in humans and various other mammals. They are responsible for giardiasis and cryptosporidiosis. Their transmission forms are cysts and oocysts, respectively. They are usually excreted in high numbers in the feces of infected hosts and can contaminate water, soil, fruits and vegetables. Whereas *Toxoplasma gondii* responsible agent for toxoplasmosis, can be transmitted via oocysts exclusively excreted in feces of contaminated felids, definitive host of the parasite, or cysts shed in other animals' organs.

The consumption of contaminated raw fruits and vegetables is considered as a vehicle of transmission of 12 out of 24 food-borne parasites worldwide^1^ and among them oocysts for *Cryptosporidium* spp. and *T. gondii*, and cysts for *G. duodenalis*^2^.

Several outbreaks related to vegetables or water contaminated with these protozoan parasites have been reported since the 1990s 3,4. Looking for a potential foodborne contamination, various studies have investigated the contamination of fresh vegetables sold for consumption with protozoan parasites and the parasitic rates varied from a country to another and even inside one country.

Nowadays, there is a global increase of the tendency for eating raw or slightly cooked vegetables to preserve their taste and heat labile nutrients that may increase the risk of foodborne infections^5^. In addition, the increasing exchange all over the world of fresh food preserved in refrigerated conditions, exposes a wider population to infection as the transmission stages of many parasites are stable especially in refrigerated conditions 6. Due to their morbidity, foodborne infectious diseases are considered as public health problems and raise significant economic impacts. The World Health Organization estimates that every year one third of the world's population suffers from foodborne diseases and that diarrhea, which is one of the main symptom caused by the ingestion of food and water contaminated with microorganisms, causes 2.1 million deaths worldwide^7^.

In Morocco, there is a lack of data about contamination rate of *Cryptosporidium spp.*, *G. duodenalis* and *T. gondii* in vegetables sold for local consumption. Herein we describe the results of a survey undertaken between March 2017 and January 2018 to determine the occurrence of transmission stages of these pathogens in some leafy greens and root vegetables commercially available in Marrakech.

## Material and methods

### Study area

Marrakech is the fourth largest city of the Kingdom of Morocco, with a latitudinal position of 31°38′02″ North and 7°59′59″ West. It is at a height of about 457m above sea level and has an arid and warm Mediterranean climate, characterized by low and variable rainfall, a high average temperature, with strong monthly and daily differences, low humidity and strong evaporation^8^. It remains subject to the influences of the Atlantic Ocean and that of the very high regions of the High Atlas. The average annual precipitation in Marrakech, calculated over the year 2017, was 122 mm. Their annual distribution contrasts with a rainy season from October to April, with maxima in November-December and March-April, and a period of almost absolute drought, in summer, accentuated by drying winds. The annual average temperature was 23.1 °C, ranging from an average of 11.6 °C in January to 31 °C in August 9.

### Vegetables sources

Vegetables were purchased from three different markets in Marrakech to cover the majority of supply sources of fresh vegetables for the population of the city. These markets were: i) the wholesale vegetable market based in the city and organized in sheds or sales areas, ii) a supermarket situated in the city center and iii) rural retailers organized in an open area of Ghmate village located in a peripheral zone at 30 km from Marrakech city center. These markets can be supplied by local and/or imported vegetables from different regions of Morocco.

### Samples preparation and analysis

This study included carrot, coriander, lettuce, parsley and radish. Their choice depended on their availability and form of consumption; raw and/or sparingly cooked. Vegetable samples were randomly collected per month from each market, from March 2017 to January 2018. A total of 132 samples were obtained from the three markets in Marrakech (Table I). They were shopped into clean plastic bags, transported to the laboratory and analyzed as followed: damaged leaves, stems and roots were discarded and the size of vegetable samples was harmonized at 200 g. Each sample was directly washed in a crystallizer with distilled water (volumes of 1 l and 300 ml were used respectively for leafy greens and root vegetables), under horizontal agitation (150 agitation/min, amplitude 25 mm) for 40 min. The sample was removed, and the washing water was left overnight for sedimentation to take place. The top layer was then discarded, and the remaining water (100 ml) was centrifuged at 855 *g* for 30 min. The supernatant was discarded and the pellet (200 µl) was divided into two portions: 100 µl for microscopic examination (x10, x40) using modified Ziehl-Neelsen staining and 100µl for quantitative polymerase chain reaction (qPCR) assay.

**Table I:** Details of vegetables sampling in Marrakech, Morocco

	Seasons	Spring 2017				Summer 2017				Autumn 2017				Winter 2018				Total
Vegetables	Origin	SM	WM	RM	Total	SM	WM	RM	Total	SM	WM	RM	Total	SM	WM	RM	Total	
**Carrot**	Number of analyzed samples	3	3	3	**9**	2	2	2	**6**	3	3	3	**9**	2	2	2	**6**	**30**
**Coriander**	Number of analyzed samples	3	3	3	**9**	2	2	2	**6**	3	3	3	**9**	2	1	2	**5**	**29**
**Lettuce**	Number of analyzed samples	3	3	3	**9**	2	2	2	**6**	3	3	2	**8**	2	2	1	**5**	**28**
**Parsley**	Number of analyzed samples	3	4	3	**10**	2	2	2	**6**	3	2	3	**8**	2	1	2	**5**	**29**
**Radish**	Number of analyzed samples	3	3	0	**6**	0	1	0	**1**	3	2	0	**5**	2	2	0	**4**	**16**

SM: Supermarket, WM: Wholesale market, RM: Rural market

### Modified Ziehl-Neelsen staining

Samples smears were made from the pellets (100 µl) and were air-dried. The staining involved the following steps: a) fixation in ethanol for 5 minutes, b)staining with 1% carbol-fuchsin for 60 minutes, c) rinsing thoroughly in tap water, d) decolorizing in acid alcohol (2% H^2^SO^4^ in ethanol) for 20 seconds, e)rinsing thoroughly in tap water, f) counter staining with 5% malachite green for 1 minute, g), rinsing thoroughly and air drying. Microscopic examination was then performed: *Cryptosporidium spp*. oocysts appeared as bright rose-pink spherules on pale green background.

In the present study, we didn't use microscopic observation for *G. duodenalis* cysts and *T. gondii* oocysts.

### PCR assay

Pellets (100 µl) were submitted to physical treatments in order to break the walls of (oo)cysts using the Fast-Prep-24 5G™ High Speed (MP Biomedicals). Then, DNA was extracted and purified using FastDNA™ SPIN Kit for Soil (MP Biomedicals) as recommended by the manufacturer. The DNA extracts (100 µl) were stored at -20°C until analysis by qPCR. Specific DNA amplification of *C. parvum*/*hominis, G. duodenalis* and *T. gondii* were performed in a QuantStudio3 Real time PCR system (Thermo Fisher). DNA Templates (5 µl) were added to a reaction mixture (20 µl) containing H^2^0, iQ™ Supermix (Bio-Rad), 400 nM of each primer and 200 nM of probe (Probes, primers and targets are summarized in Table II). A no-template control was added. qPCR was divided into 2 steps; the DNA denaturation at 95 °C for 3 min and amplification through 40 cycles of 15 s at 95 °C and 1 min at 60 °C. PCR reaction was performed in duplicates and Cq (quantification cycle) values were averaged. The Cq value corresponds to the cycle number at which the fluorescence exceeds a fixed threshold and allows the quantification of the amount of the target DNA. In internal conditions, we have considered positive samples when the Cq values were inferior to 40, for both DNA deposits (data not shown). Moreover, in case the DNA was detected only in one deposit, a second qPCR analysis was performed again using the same procedure. In this case, the positivity limit was set at 2/4 positive deposits, at least.

**Table II:** Probes, primers and targets used for the detection of *C. parvum/hominis, G. duodenalis* and *T. gondii* in vegetables from Marrakch, Morocco (as described previously by Palos Ladeiro et al. (2014)^10^)

Parasite	Target	Primers	Sequence of primers (5′-3′)	Probe	Sequence of probe (5′-3′)	Reference
*C. parvum/* *hominis*	**DNA J like** **protein**	Crypto-F	CGCTTCTCTAGCCTTTTCATGA	Crypto-P	FAM-CCAATCACAGAATCATCAGAATCGACTGGTATC-BHQ1	Fontaine and Guillot, (2002)^11^

		Crypto-R	CTTCACGTGTGTTTGCCAAT			

*G.* *duodenalis*	**16S-like** **rRNA**	G-80F	GACGGCTCAGGACAACGGTT	GP-105T	FAM-CCCGCGGCGGTCCCTGCTAG-BHQ1	Verweij et *al.* (2004)^12^

		G-127R	TTGCCAGCGGTGTCCG			

*T.* *gondii* strain RH	**Repeat** **region** **AF487550.1**	Toxo-F	AGAGACACCGGAATGCGATCT	Toxo-P	FAM-ACGCTTTCCTCGTGGTGATGGCG-BHQ1	Reischl et *al.* (2003)^13^

		Toxo-R	CCCTCTTCTCCACTCTTCAATTCT			

## Results

Positive samples are reported in Table III, considering the vegetable types and their sampling origin from Marrakech, in Morocco.

**Table III:** Details of qPCR analysis results, for the detection of *G. duodenalis* and *T. gondii* in fresh vegetables collected from different markets in Marrakech

Sample type	Sampling origin	No of samples	Total of positive samples*	No of positive samples/ Total No analyzed					
				2 deposits/2**		3 deposits/4***		2 deposits/4***	
				*T. gondii*	*G. duodenalis*	*T. gondii*	*G. duodenalis*	*T. gondii*	*G. duodenalis*
**Carrot**	Supermarket	10	0/10	0/10	0/10	0/10	0/10	0/10	0/10
	Wholesale market	10	2/10	0/10	0/10	0/10	0/10	2/10	0/10
	Rural market	10	1/10	0/10	0/10	0/10	0/10	1/10	0/10
**Coriander**	Supermarket	10	1/10	0/10	0/10	0/10	0/10	0/10	1/10
	Wholesale market	9	1/9	0/9	0/9	0/9	0/9	1/9	0/9
	Rural market	10	6/10	2/10	2/10	1/10	1/10	0/10	0/10
**Lettuce**	Supermarket	10	1/10	0/10	0/10	1/10	0/10	0/10	0/10
	Wholesale market	10	0/10	0/10	0/10	0/10	0/10	0/10	0/10
	Rural market	8	2/8	1/8	1/8	0/8	0/8	0/8	0/8
**Parsley**	Supermarket	10	5/10	4/10	0/10	0/10	0/10	1/10	0/10
	Wholesale market	9	5/9	2/9	1/9	1/9	0/9	1/9	0/9
	Rural market	10	3/10	2/10	0/10	1/10	0/10	0/10	0/10
**Radish**	Supermarket	8	1/8	0/8	0/8	0/8	0/8	1/8	0/8
	Wholesale market	8	0/8	0/8	0/8	0/8	0/8	0/8	0/8
	Rural market	0	0	0	0	0	0	0	0

* positive samples: Cq mean value inferior to 40.

** positive samples for both DNA deposits after first qPCR.

*** positive samples for one DNA deposit after first qPCR and subjected to a second qPCR analysis considering four DNA deposits.

*C. parvum*/*hominis* were not detected in any vegetable sample, using qPCR technique, whereas 3% (4/132) samples were positive for *Cryptosporidium spp.*, using modified Ziehl-Neelsen staining: 2/29 in parsley, 1/28 and 1/29 in lettuce and coriander, respectively.

*G. duodenalis* was detected, by qPCR, in 13.8% (4/29) samples of coriander, although only one positive sample was observed for lettuce and parsley each; while all carrot and radish samples were found to be free of this parasite.

The most detected parasite, using qPCR, was *T. gondii*, with the highest prevalence rate 41.4% (12/29) in parsley, followed by coriander 13.8% (4/29), carrot 10% (3/30), lettuce 7.1% (2/28) and radish 6.3% (1/16).

Of the 132 samples of vegetables analyzed by qPCR, the overall rate of protozoan parasites was 21.21% (28/132); 22 samples were found to be contaminated with *T. gondii*, six with *G. duodenalis*, whereas *C. parvum*/*hominis* were not detected. Among all examined samples, parsley showed the highest parasitic prevalence 45% (13/29), followed by coriander 27.6% (8/29), lettuce 11% (3/28), carrot 10% (3/30) and radish 6.3% (1/16).

**Figure 1:**
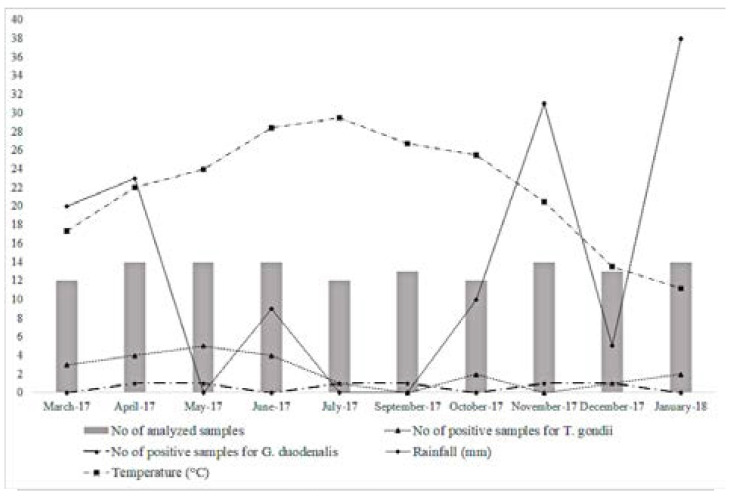
Distribution of positive samples for *T. gondii* and *G. duodenalis*, considering the climatological data of Marrakech, from March 2017 to January 2018

Figure 1 illustrates the distribution of positive samples for *T. gondii* and *G. duodenalis* considering the climatological data (rainfall and temperature) recorded in Marrakech, in the period of this study. It shows that the presence of both *T. gondii* and *G. duodenalis* can be affected by both dry and cold conditions, however, it could not be exclusively influenced by the climatological variations as other factors seem contributing to the survival and attachment of some protozoan parasites in vegetables.

## Discussion

The present survey investigated the presence of pathogenic protozoan parasites, namely *Cryptosporidium spp.*, *G. duodenalis* and *T. gondii*, in leafy greens and root vegetables. It showed that marketed vegetables in Marrakech are contaminated by one or more protozoan pathogens.

Microscopy and molecular techniques allowed the detection of *Cryptosporidium spp.*, *G. duodenalis* and *T. gondii*, with an overall prevalence of 24% (32/132). The most frequent protozoan was *T. gondii* followed by *G. duodenalis* and *Cryptosporidium spp*. (Table III).

*Cryptosporidium* **oocysts detection:**

There was a difference between the rate of *Cryptosporidium spp.* obtained using Modified Ziehl-Neelsen staining and qPCR assay. Indeed, 3% (4/132) of analyzed samples were positive for this parasite by microscopy while all of them were negative by qPCR. In addition, these findings were confirmed by immuno-chromatography (data not shown). This could be explained by the hypothesis that *Cryptosporidium spp*. oocysts observed by microscopy do not belong neither to *C. parvum* nor to *C. hominis*, that are targeted by the qPCR used in our study.

This study showed a low level of contamination of leafy greens and root vegetables with *Cryptosporidium spp.* (parsley 2/29, lettuce 1/28, coriander 1/29, carrot 0/30 and radish 0/16) in Marrakech. Higher rates of this pathogen were recorded in Alexandria 14 (Egypt), in samples of lettuce 26/60 and parsley 20/60; as in Arba Minch^15^ (Ethiopia), where 2/23 lettuce and 7/62 carrot samples were contaminated. On the other hand, the examination of vegetables samples collected within two Egyptian governorates (Mnia and Assiut)^16^ revealed lower rates of contamination (lettuce 0/73 and parsley 3/88), noting that the studies used microscopic examination. Macarisin et al. (2010)^17^ demonstrated that *Cryptosporidium* oocysts were capable of strongly adhering to spinach plants after contact with contaminated water and were also internalized within the leaves, that made this pathogen resist to washing. In fact, samples processing usually involved among other steps washing vegetables that may result in preventing a better recovery of oocysts internalized in vegetable leaves. So, the contamination of vegetables with *Cryptosporidium* oocysts reported among the cited studies could be underestimated.

*Giardia* **cysts detection:**

In our work, the parasitic contamination with *G. duodenalis* was evaluated at 4.5% by qPCR assay (coriander 4/29, lettuce 1/28, parsley 1/29, carrot 0/30 and radish 0/16). Previously, Amahmid et al. (1999)^18^ evaluated the presence of *Giardia* cysts in crops irrigated with three types of water; raw wastewater, treated wastewater and fresh water, in Marrakech. This study confirmed that the use of raw wastewater for irrigation leads to parasitic contamination, indeed, 4/9 coriander, 3/9 carrot and 4/9 radish samples were positive for *G. duodenalis*, whereas crops irrigated with treated wastewater and fresh water were free from contamination. Our finding was in agreement with another one in Tripoli^19^, Libya, that found 1/27 of lettuce samples contaminated with this pathogen. As well as in Egypt^14^ where 9/60 of lettuce and 3/60 of parsley samples were contaminated. Lower rates were reported in lettuce (0/23 15, 2/73 16) and parsley (0/88 16), while carrot contamination was slightly higher (4/62 15 and 3/41 16) in Ethiopia and Egypt, respectively.

*Toxoplasma* **oocysts detection:**

The contamination with *T. gondii* detected in this study was 16.6% using qPCR, it was the most prevalent parasite contaminating vegetables (parsley 12/29, coriander 4/29, carrot 3/30, lettuce 1/28 and radish 1/16). Few studies have been conducted to investigate the presence of this parasite in fresh produce, especially in Africa. The study conducted in Egypt 16 showed that 3/73 lettuce, 3/88 parsley and 5/41 carrot samples were found positive for *T. gondii*, using microscopy.

Although there are few reported studies in Africa on the prevalence of *Cryptosporidium, Giardia* and *Toxoplasma* in fresh produce, there is still a lack of data in Morocco regarding the presence of these pathogens in marketed vegetables. So, we wanted to explore the presence of these parasites using highly sensitive techniques.

Our finding revealed that leafy greens were the most contaminated compared to root vegetables. Indeed, parsley showed the highest parasitic rate (45%), followed by coriander (27,6%), lettuce (11%), carrot (10%) and radish (6,3%). This difference could be related to the fact that leafy greens as parsley and coriander have dense foliage, while lettuce has broad leaves and large surface areas providing a large contamination surfaces, which are in direct contact with contaminated soil and water, hence their high contamination rates^20^. In addition, the surface structure could also affect the parasitic attachment. Indeed, vegetables with rough surfaces are more susceptible for contamination than vegetables with smooth surfaces like radish and carrot.

The difference between our results and the parasitic prevalence reported by the other studies is mainly due to:

-The detection methods including the elution of protozoan parasites from vegetable matrices, their concentration and identification. These three steps affect the recovery of protozoan parasites. For this purpose, various elution procedures have been evaluated for the elution of protozoan parasites from vegetables matrices. Indeed, Cook et al. (2006)^21^ evaluated different elution buffers (extractants) (i.e. PBS, pH 7.2; 0.1 M tricine, pH 5.4; 1% lauryl sulfate; 1 M Glycine, pH 5.5) for the detection of *C. parvum* in lettuce, as they tested various elution procedures (i.e. stomaching, pulsification, rolling, orbital shaking), coupled to immunofluorescence assay (IFA). They demonstrated that the percentage of recovered oocysts differed depending on the extractants and the physical extraction methods. They proved that a Glycine buffer ‘1M Glycine’ with an optimal pH of 5.5, was the cheapest and effective buffer compared to the other ones, especially when it was used with stomaching.

Otherwise, Shields et al. (2012)^22^ compared various buffers as deionized water, 1 M Glycine, pH 5.5, a detachment solution, and 0.1% Alconox®, to recover *C. parvum* and *Cyclospora cayetanensis* from lettuce, herbs and raspberries; there was no significant difference between the deionized water and 1 M glycine, pH 5.5, while Alconox gave the best recovery rate.

On the other hand, Shapiro et al. (2019)^23^ described a method for simultaneous detection of *C. parvum, G. duodenalis, T. gondii* and *C. cayetanensis*. They found that leaf-washing followed by a multiplex-PCR allowed higher recoveries and more consistent detection of parasites compared to stomaching processing.

In fact, the use of deionized water for protozoan parasites elution, in our study, was based on its low cost and effectiveness.

As the elution step, the concentration of protozoan parasites from eluates was performed using different procedures as centrifugation 17–24–25, that could be followed either by purification (using sucrose flotation for example)^26^–^27^ or by immunomagnetic separation (IMS) 6–28–29. Although, among the three concentration procedures, centrifugation was more commonly used since it is a direct, simple, rapid and non-expansive technique. Hence its use in this study.

Moreover, the final step that aims to detect protozoan parasites in vegetables was conducted using various methods with different sensibilities. Among the described methods, acid fast stains as modified Ziehl-Neelsen staining were used in the identification of *Cryptosporidium*. It is considered as one of the most widely used techniques for *Cryptosporidium* identification^16^–^25^–^26^–^30^–^31^. Another method is the IFA which is commonly used for the detection of parasites (oo)cysts in various vegetable matrices, and it is the method recommended by ISO (ISO 18744 method for the detection of *Cryptosporidium* and *Giardia* in leafy greens and berry fruits^32^). However, this method is time consuming, requires an expensive technology and a microscopy expertise^33^. Otherwise, other studies described molecular approaches that allow the detection of protozoan parasites in vegetables (qPCR^27^–^28^, multiplex-PCR^23^), and the determination of their viability (reverse transcriptase qPCR^34^). Herein, we used qPCR which is a rapid and sensitive method. In fact, there are a multitude of detection methods that depend on the vegetable types and the targeted parasites, hence the importance of defining a standardized method for the simultaneous detection of pathogens as *C. parvum, G. duodenalis* and *T. gondii* in vegetable matrices.

-The geographical location, that is related to the development level of the different countries, which conditions the level of application of the good hygiene practices during the irrigation and the harvesting of vegetables, as it can be due to climate changes^35^.

Our study area can be considered as a dry region since the average annual precipitation calculated over the year 2017, was 122 mm. The dry conditions affect the survival of *T. gondii* oocysts as demonstrated by Lélu et al. (2012)^36^, where the proportion of oocysts surviving in soil after 100 days was 7,4% in dry conditions (281 mm of precipitation per year) and 43,7% in damp conditions (3648 mm of precipitation per year). This finding was consistent with our results since the overall rate of contaminated vegetables with *T. gondii* was 16.6% (21/132). Our samples presented a low rate of contamination, since 11/132 of samples were positive for both qPCR replicates, while 11/132 of samples were confirmed to be positive after a second qPCR analysis: 7/132 of samples were positive with 2/4 replicates and 4/132 were positive with 3/4 replicates. Otherwise, *G. duodenalis* cysts were present in 4.5% (6/132) of analyzed samples, four samples were positive for both qPCR replicates and two were confirmed positive after a second analysis.

Dry conditions may lead to reduced water availability and compaction, contributing to increased runoff after rainfall 37.

Moreover, in dry climate, there is an increased use of alternative water sources as wastewater for irrigation, increasing the risk of vegetables contamination. The presence of these waterborne parasites may mainly be related to irrigation water that could be a vehicle for these parasites considering that untreated raw water could have been used for irrigation as well as chlorinated water, noting that oocysts and cysts are resistant to chlorination treatment.

In addition, the contamination of vegetables with *T. gondii* oocysts may occur in farms via culture in contaminated soil with cat feces containing *T. gondi*i oocysts. Considering the climatological data (Rainfall and temperature) recorded in Marrakech, in the period of this study (Figure 1), the distribution of positive samples for both *T. gondii* and *G. duodenalis* could not be exclusively influenced by rainfall and temperature variations. Although excess precipitation may result in increased runoff and turbidity and decreased effectiveness of water treatment^37^, dry periods may induce increased use of irrigation water sources eventually contaminated especially when farmers are not aware of hygiene and sanitation practices.

The presence of parasites and vegetables origin (Table III) seemed to be independent, that could be due to the lack of traceability and consequently different sampling points could be supplied by the same vegetable source. The presence of pathogenic protozoan parasites in marketed fresh vegetables in Marrakech may represent a health risk for consumers, leading to parasitic diseases. This was already illustrated by the study of Ait Melloul et al. (2010) in Azzouzia^38^ (the wastewater spreading area of Marrakech city), with 39% of giardiasis infections observed among children living in areas where untreated water was used for irrigation. This finding joined other Moroccan epidemiological data such as El Fatni et al. (2014) in Tetouan^39^, El Guamri et al. (2011) in Kenitra^40^ and Habbari et al. (2000) in Beni Mellal^41^, where the rates of giardiasis were 20% of 673 examined children, 14% and 5.1%, respectively. On the other hand, few studies have been carried out in Morocco to determine the seroprevalence of toxoplasmosis, for example 33.3% of women in Marrakech^42^, and 50.6% of pregnant women in Rabat^43^ were infected by *T. gondii*. Whereas, there was no epidemiological studies done in Morocco to determine the prevalence of *Cryptosporidium spp*. among human population.

## Conclusion

The present study is the first one in Morocco on the prevalence of protozoan parasites in vegetables. Our results show that marketed fresh vegetables in Marrakech and intended for human consumption are contaminated with the three parasites investigated in the present study. This exposes the consumer to the risk of contamination although vegetables are not the only vehicle of parasitic transmission to humans (waterborne transmission for example). On the other hand, the lack of traceability of vegetables in the study region as well as the lack of investigations of the prevalence of these pathogens in both humans and fresh produce, prevent us from establishing an epidemiological map of these pathogens and making a link between the presence of parasites and parasitosis. Several studies in Morocco are necessary to i) consolidate our data on described protozoan and ii) targeting other protozoan, to determine outbreaks due to foodborne parasites as *Cyclospora cayetanensis*.

## References

[1] Robertson LJ, Van der Giessen JWB, Batz MB, Kojima M, Cahill S (2013). Have Foodborne Parasites Finally Become a Global Concern?. Trends Parasitol.

[2] Slifko TR, Smith HV, Rose JB (2000). Emerging parasite zoonoses associated with water and food. Int J Parasitol.

[3] Baldursson S, Karanis P (2011). Waterborne transmission of protozoan parasites: review of worldwide outbreaks - an update 2004–2010. Water Res.

[4] Robertson LJ, Chalmers RM (2013). Foodborne cryptosporidiosis: is there really more in Nordic countries?. Trends Parasitol.

[5] FAO/WHO [Food and Agriculture Organization of the United Nations/World Health Organization] (2014). Multicriteria-based ranking for risk management of foodborne parasites. Microbiological Risk Assessment Series, 23.

[6] Robertson LJ, Gjerde B (2001). Occurrence of parasites on fruits and vegetables in Norway. Food Prot.

[7] WHO/FAO (2003). Diet, nutrition, and the prevention of chronic diseases: report of a WHOFAO Expert Consultation; [Joint WHO-FAO Expert Consultation on Diet, Nutrition, and the Prevention of Chronic Diseases, 2002, Geneva, Switzerland].

[8] Boutayeb K (1988). Caractérisation hydrochimique des eaux souterraines du Haouz (Marrakech).

[9] Ait Boughrous A (2007). Biodiversité, écologie et qualité des eaux souterraines de deux régions arides du Maroc : le Tafilalet et la région de Marrakech.

[10] Palos Ladeiro M, Aubert D, Villena I, Geffard A, Bigot A (2014). Bioaccumulation of human waterborne protozoa by zebra mussel (*Dreissena polymorpha*): Interest for water biomonitoring. Water Res.

[11] Fontaine M, Guillot E (2002). Development of a TaqMan quantitative PCR assay specific for *Cryptosporidium parvum*. FEMS Microbiol Lett.

[12] Verweij JJ, Schinkel J, Laeijendecker D, van Rooyen MAA, van Lieshout L, Polderman AM (2003). Real-time PCR for the detection of *Giardia lamblia*. Mol Cell Probes.

[13] Reischl U, Bretagne S, Kruger D, Ernault P, Costa JM (2003). Comparison of two DNA targets for the diagnosis of toxoplasmosis by real-time PCR using fluorescence resonance energy transfer hybridization probes. BMC Infect Dis.

[14] El Said Said D (2012). Detection of parasites in commonly consumed raw vegetables. Alexandria J Med.

[15] Alemu G, Mama M, Misker D, Haftu D (2019). Parasitic contamination of vegetables marketed in Arba Minch town, southern Ethiopia. BMC Infect Dis.

[16] Ahmad SO, El Fadaly HA, Zaki MS, Barakat AMA (2016). Incidence of Zoonotic Parasites in Egyptian raw vegetable salads. Life Sci.

[17] Macarisin D, Bauchan G, Fayer R (2010). *Spinacia oleracea* L. Leaf stomata harboring *Cryptosporidium parvum* oocysts: a potential threat to food safety. Appl Environ Microbiol.

[18] Amahmid O, Asmama S, Bouhoum K (1999). The effect of wastewater reuse in irrigation on the contamination level of food crops by *Giardia* cysts and *Ascaris* eggs. Int J Food Microbiol.

[19] Abougrain AK, Nahaisi MH, Madi NS, Saied MM, Ghenghesh KS (2010). Parasitical contamination in salad vegetables in Tripoli-Libya. Food control.

[20] Eraky MA, Mostafa Rashed S, El-Sayed Nasr M, Mohammed Salah El-Hamshary A, Salah El-Ghannam A (2014). Parasitic contamination of commonly consumed fresh leafy vegetables in Benha, Egypt. Parasitol Res.

[21] Cook N, Paton CA, Wilkinson N, Nichols RAB, Barker K, Smith HV (2006). Towards standard methods for the detection of *Cryptosporidium parvum* on lettuce and raspberries. Part 1: development and optimization of methods. Int J Food Microbiol.

[22] Shields JM, Lee MM, Murphy HR (2012). Use of a common laboratory glassware detergent improves recovery of *Cryptosporidium parvum* and Cyclospora cayetanensis from lettuce, herbs and raspberries. Int J Food Microbiol.

[23] Shapiro K, Kim M, Rajal VB, Arrowood MJ, Packham A, Aguilar B, Wuertz S (2019). Simultaneous detection of four protozoan parasites on leafy greens using a novel multiplex PCR assay. Food Microbiol.

[24] Lass A, Pietkiewicz H, Szostakowska B, Myjak P (2012). The first detection of *Toxoplasma gondii* DNA in environmental fruits and vegetables samples. Eur J Clin Microbiol Infect Dis.

[25] Avazpoor M, Taqi Yousefipoor M, Dusty M, Mehdipour M, Seifipour F, Gholami Z (2015). Determination of the level of parasitic infection (*Cryptosporidium* and *Giardia*) of the vegetables marketed in Ilam city. Environ Health Eng Manag.

[26] El Sherbini GT, Osman Hany Kamel N, Geneedy MR, Temsah AG (2016). A comparative study of the occurrence of *Cryptosporidium parvum* oocysts found on fresh fruits and vegetables sold in supermarkets and open-aired markets. Int J Curr Microbiol Appl Sci.

[27] Lalonde LF, Gajadhar AA (2016). Detection of *Cyclospora cayetanensis, Cryptosporidium* spp., and *Toxoplasma gondii* on imported leafy green vegetables in Canadian survey. Food Waterborne Parasitol.

[28] Hong S, Kim K, Yoon S, Park WY, Sim S, Yu JR (2014). Detection of *Cryptosporidium parvum* in Environmental Soil and Vegetables. J Korean MedSci.

[29] Utaaker KS, Huang Q, Robertson LJ (2015). A reduced-cost approach for analyzing fresh produce for contamination with *Cryptosporidium* oocysts and/or *Giardia* cysts. Food Res Inter.

[30] Calvo M, Carazo M, Arias ML, Chaves C, Monge R, Chinchilla M (2014). Prevalence of *Cyclospora* sp, *Cryptosporidium* sp, *Microsporidia* and fecal coliform determination in fresh fruit and vegetables consumed in Costa Rica. Arch Latinoam Nutr.

[31] Maikai BV, Baba-Onoja EBT, Elisha IA (2013). Contamination of raw vegetables with *Cryptosporidium* oocysts in markets within Zaria metropolis, Kaduna State, Nigeria. Food Control.

[32] (2016). Microbiology of the food chain—detection and enumeration of *Cryptosporidium* and *Giardia* in fresh leafy green vegetables and berry fruits.

[33] Ahmed SA, Karanis P (2018). An overview of methods/techniques for the detection of *Cryptosporidium* in food samples. Parasitol Res.

[34] Hohweyer J, Cazeaux C, Travaillé E, Languet E, Dumètre A, Aubert D (2016). Simultaneous detection of the protozoan parasites *Toxoplasma, Cryptosporidium* and *Giardia* in food matrices and their persistence on basil leaves. Food Microbiol.

[35] Fletcher SM, Stark D, Harkness J, Ellis J (2012). Enteric protozoa in the developed world: a public health perspective. Clin Microbiol Rev.

[36] Lélu M, Villena I, Dardé ML, Aubert D, Geers R, Dupuis E (2012). Quantitative estimation of the viability of *Toxoplasma gondii* oocysts in soil. App Environ Microbiol.

[37] Charron DF, Thomas MK, Waltner-Toews D, Aramini JJ, Edge T, Kent RA (2004). Vulnerability of waterborne diseases to climate change in Canada: a review. J Toxicol Environ Health.

[38] Ait Melloul, Amahmid O, Hassani L, Bouhoum K (2010). Health effect of human wastes use in agriculture in El Azzouzia (the wastewater spreading area of Marrakesh city, Morocco). Int J Environ Health Res.

[39] El Fatni C, Olmo F, El Fatni H, Romero D, Rosales MJ (2014). First genotyping of *Giardia duodenalis* and prevalence of enteroparasites in children from Tetouan (Morocco). Parasite.

[40] EL Guamri Y, Belghyti D, Barkia A, Tiabi M, Aujjar N, Achicha A (2011). Parasitic infection of the digestive tract in children in a Regional Hospital Center in Gharb (Kenitra, Morroco): Some epidemiological features. East Afr J Public Health.

[41] Habbari K, Tifnouti A, Bitton G, Mandil A (2000). Intestinal parasitosis and environmental pollution: 1343 pediatric cases in Beni-Mellal, Morocco. Tunis Med.

[42] Biava MF, Jana M, EL Mansouri A, Percebois G (1983). Etude séro-épidémiologique de la toxoplasmose à Marrakech. Med Mal Infect.

[43] El Mansouri B, Rhajaoui M, Sebti F (2007). Séroprévalence de la toxoplasmose chez les femmes enceintes à Rabat. Bull Soc Pathol Exot.

